# The seminal acrosin‐inhibitor ClTI1/SPINK2 is a fertility‐associated marker in the chicken

**DOI:** 10.1002/mrd.23153

**Published:** 2019-04-29

**Authors:** Aurore Thélie, Sophie Rehault‐Godbert, Jean‐Claude Poirier, Marina Govoroun, Sophie Fouchécourt, Elisabeth Blesbois

**Affiliations:** ^1^ PRC INRA, CNRS, IFCE, Université de Tours Nouzilly France; ^2^ BOA INRA Nouzilly France

**Keywords:** acrosin, chicken, male fertility, protease inhibitor, seminal plasma

## Abstract

The seminal plasma is a very complex fluid, which surrounds sperm in semen. It contains numerous proteins including proteases and protease inhibitors that regulate proteolytic processes associated with protein activation and degradation. We previously identified a seminal protein, chicken liver trypsin inhibitor 1 (ClTI‐1) over expressed in semen of roosters with high fertility, suggesting a role in male fertility. In the present study, we showed that *ClTI‐1* gene is actually *SPINK2.* Using normal healthy adult roosters, we showed that SPINK2 amount in seminal plasma was positively correlated with male fertility in chicken lines with highly contrasted genetic backgrounds (broiler and layer lines). Using affinity chromatography combined to mass spectrometry analysis and kinetic assays, we demonstrated for the first time that two chicken acrosin isoforms (acrosin and acrosin‐like proteins) are the physiological serine protease targets of SPINK2 inhibitor. SPINK2 transcript was overexpressed all along the male tract, and the protein was present in the lumen as expected for secreted proteins. Altogether, these data emphasize the role of seminal SPINK2 Kazal‐type inhibitor as an important actor of fertility in birds through its inhibitory action on acrosin isoforms proteins.

## 1 INTRODUCTION

Understanding the mechanisms involved in animal sperm quality is important to improve fertilization success and to face major challenges including improvement in farming, animal selection, conservation of genetic resources, and biodiversity. Seminal plasma is a very complex fluid composed of secretions from testis and male genital tract. In addition to previous biochemical studies, proteomic approaches allowed the identification of numerous seminal plasma proteins in the chicken and mammalian species (Atikuzzaman et al., 2017; Borziak, Álvarez‐Fernández, Karr, Pizzari, & Dorus, 2016; Brito et al., 2018; Camargo, Intasqui, & Bertolla, 2018; Druart et al., 2013; Intasqui et al., 2016; Labas et al., 2015; Perez‐Patino et al., 2018; Samanta, Parida, Dias, & Agarwal, 2018). Proteomics profiles reflect sperm functional traits (Intasqui et al., 2016) and highly differ between species. A significant number of seminal proteases and protease inhibitors are suspected to participate in the regulation of maturation/activation, release, and protection of sperm (Cesari, Monclus Mde, Tejon, Clementi, & Fornes, 2010; Gurupriya et al., 2014; Laflamme & Wolfner, 2013). In many species, acrosin is an essential trypsin‐like serine protease stored in the sperm acrosome as an inactive zymogen form, proacrosin, before its activation by limited proteolysis that releases the active acrosin at the time of fertilization (Bozzola et al., 1991; Slowinska, Olczak, Liszewska, Watorek, & Ciereszko, 2010). In mammals, acrosin is considered to play an essential role in fertilization, by contributing to the recognition and the enzymatic digestion of the zona pellucida (Mao & Yang, 2013).

Bird reproduction differs from mammals by many aspects. It combines a highly specialized internal fertilization system with specific storage of sperm in the female tract (sperm storage tubules [SST]), physiological polyspermy, specific relationships between sperm and oocyte perivitelline layer, oviparity with telolecithal egg but very rapid divisions of the zygote in the female tract before oviposition. All adaptations making easier the “adaptive freedom” of flying animals. Moreover, bird sperm biology faces a spermatogenesis producing a high number of sperm (billions in the chicken) in a short time (12 days in the chicken), a lack of accessory glands in the male tract, and a post gonadic process in the female tract that is much longer (3 weeks in the chicken, 2–3 months in the turkey) where sperm evolve in highly different environments depending on the stage of albumen/shell secretions (reviewed by Blesbois, 2018). In birds, acrosin is assumed to be involved in the interaction of sperm with the inner perivitelline layer that surrounds the oocyte at the earliest step of the fertilization process (Etches, Clark, Toner, Liu, & Gibbins, 1996; Lemoine, Grasseau, Brillard, & Blesbois, 2008; Slowinska et al., 2010; Slowinska, Liszewska, Dietrich, & Ciereszko, 2012).

The secretion of proteases as zymogens (i.e., proacrosin is the zymogen form of acrosin) is a way to preserve proteins from degradation and to prevent their early activation. An additional way to limit the excessive proteolysis is the presence of specific natural inhibitors, such as Kazal‐type protease inhibitors of the SPINK family (Laskowski & Kato, 1980). SPINK proteins contain at least a kazal domain and six consensus cysteines forming a 1–5/2–4/3–6 disulfide bound pattern (Kazal, Spicer, & Brahinsky, 1948; Rawlings, Tolle, & Barrett, 2004) and are trypsin and trypsin‐like proteases inhibitors (Laskowski & Kato, 1980). Some of them are expressed in the reproductive tract of mammals (Cesari et al., 2010; Laflamme & Wolfner, 2013; Le Magueresse‐Battistoni, 2007; Moritz, Lilja, & Fink, 1991; Perry, Jones, & Hall, 1993) and concentrated in the seminal plasma (Jonakova et al., 1992) where they protect sperm from proteolytic degradation (Laskowski & Kato, 1980; Perry et al., 1993). In vertebrates, only few SPINK proteins with a single domain have been identified, SPINK3 and SPINK‐L in the seminal vesicles, SPINK13 in the epididymis, and SPINK2 (also named sperm‐associated acrosin inhibitor in boar and HUSI‐II in human), in mammalian testis, epididymis, and seminal vesicles, and in carp testis (Chen, Lin, Lai, & Chen, 1998; Dietrich et al., 2017; Lin et al., 2008; Ma et al., 2013; Manaskova‐Postlerova et al., 2016; Moritz et al., 1991; Zalazar et al., 2012). SPINK3 protein binds to mice sperm, modulates sperm activity and inhibits acrosome reaction (Ou et al., 2012; Zalazar et al., 2012). SPINK‐L may inhibit mouse capacitation in vitro (Lin et al., 2008; Tseng et al., 2013). In avian species, only few members of Kazal‐type inhibitors have been characterized, essentially in egg white: ovomucoid (SPINK7) and ovoinhibitor (SPINK5; Saxena & Tayyab, 1997) and in seminal plasma: ovoinhibitor (Labas et al., 2015; Slowinska et al., 2014).

In semen, SPINK2 levels are reduced in specific pathological conditions (men azoospermia, specific reduced SPINK2 mutant mice (Kherraf et al., 2017; Lee et al., 2011; Rockett, Patrizio, Schmid, Hecht, & Dix, 2004). SPINK2 protein was recently identified as essential for acrosome biogenesis during mouse spermatogenesis (Kherraf et al., 2017). However, postgonadic SPINK2 biochemical and physiological functions in nonpathological conditions have been poorly explored and make part of the aim of the present study using the chicken model. Using an experimental line of chicken, we recently identified a seminal protein, chicken liver trypsin inhibitor 1 (ClTI‐1) that was present with variable abundance in semen of healthy roosters with contrasted fertility (Labas et al., 2015). This protein was previously purified from chicken liver and seminal plasma using a chromatography based on its trypsin inhibitor property (Kubiak, Jakimowicz, & Polanowski, 2009; Lessley & Brown, 1978) but its function as well as its target proteases remain unknown.

In the present work, we combined several complementary approaches to better decipher the post gonadic biochemical and physiological roles of the seminal ClTI‐1/SPINK2 protein in male fertility of physiologically normal, but with contrasted fertility, breeder males, and identified the physiological proteases that are actually inhibited by ClTI‐1/SPINK2.

## 2 MATERIALS AND METHODS

### 2.1 Animals and sampling

Adult birds were housed at the INRA experimental unit UE‐PEAT at Nouzilly (France). Their breeding followed the European welfare and the French Direction of Veterinary Services regulations (agreement number: C37–175‐1). Thirty‐week‐old males were obtained from commercial pedigree stocks (Hubbard, Quintin, France; Novogen, Loue, France) and housed in individual battery cages under 14L/10D photoperiod and fed with a standard diet of 12.5 MJ/day. Females used for artificial insemination (AI) were 41‐week‐old ISABROWN hens (ISA, Ploufragan, France) housed in five hens rooms under a 14L/10D photoperiod and fed a standard diet of 12.5 MJ/day, supplemented with calcium. Semen was routinely collected twice a week by massage (Burrows & Quinn, 1937). Sperm concentrations were immediately determined by light absorption of semen with a photometer (Accucell Photometer, IMV Technologies, L'Aigle, France) at a wavelength of 530 nm (Brillard & McDaniel, 1985). Individual ejaculates were then diluted 1:1 in Beltsville poultry semen extender (BPSE; Brillard & McDaniel, 1985) and then used directly to analyze semen or inseminated females. Semen was centrifuged at 600*g* for 10 min at 20°C. The supernatant corresponding to seminal plasma was centrifuged twice again at 10,000*g* for 10 min at 4°C to remove insoluble cellular debris. Pure seminal plasma was stored at −20°C until further use.

Male reproductive tract tissues were obtained from 40‐week‐old animals. For reverse transcription polymerase chain reaction (RT‐PCR), tissues were immediately frozen in liquid nitrogen, and for immunohistochemical study, the tissues were fixed in 4% paraformaldehyde (PAF) solution.

### 2.2 In vitro semen quality evaluation

Mass motility was measured as a subjective evaluation of the speed of the movement of spermatozoa in 10 µl of semen (scale 0–8), as previously described (Blesbois et al., 2008). In this motility scale, the “0” value corresponds to a total lack of movement and 8 represents whirlwinds covering 30–60% of the area. A mean of five repetitions was made per male.

Measurements of motility were evaluated by the computer‐assisted sperm analysis system with an HTM‐IVOS (Hamilton‐Thorn Motility Analyzer; IVOS, IMV Technologies), as previously described (Nguyen et al., 2014).

Sperm viability was assessed with SYBR‐14/propidium iodide fluorescent dyes (Chalah & Brillard, 1998). Sperm was diluted in Lake 7.1 buffer down to 20 × 10^6^ cells/ml and 5 µl SYBR‐14 was added before incubation for 10 min at 4°C in darkness. Afterward, 2 µl of propidium iodide was added and the incubation was further processed in the darkness for 5 min at 4°C. After incubation, sperm viability was assessed by flux cytometry measurements using a EasyCyte Guava system (Millipore, Molsheim, France).

### 2.3 In vivo fertility test

Fertility (% fertile/incubated eggs) was measured after individual intravaginal AI of 10 females/male with a dose of 100 million spermatozoa/female (Day 0). Two inseminations per female were made at a 1‐week interval for the same male. Eggs were collected from Day 2–23 post inseminations and fertility was evaluated by candling after 7 days of incubation. A mean of 250 eggs was analyzed per male.

This test is the reference test to define fertility, and allow obtaining values to establish two cohorts of animals, namely fertile and subfertile animals. Animals were, therefore, considered fertile when rates were above 70% and subfertile when rates were below 70%.

### 2.4 RNA isolation and PCR

Total RNA was extracted using TriZol reagent (Thermo Fischer Scientific, Courtaboeuf, France) according to the manufacturer's instructions. Samples were then treated with DNase (Nucleospin RNA XS Kit, Macherey Nagel, Hoerdt, France) according to manufacturer's instructions. RNA quality and concentration were determined using an Agilent 2100 Bioanalyser and the RNA 6000 labchip kit (Agilent Technologies, Les Ulis, France) following manufacturer's instructions.

For DNA synthesis, 500 ng of total RNA were denatured in the presence of a mix of oligodT (25 ng) and random hexamers (12.5 ng) for 5 min at 65°C. RT was performed at 42°C for 50 min using SuperScript II Reverse Transcriptase (Life Technologies) in the presence of dNTP (0.5 mM) and RNAsine (RNAse inhibitor, 2 U). All products were from Promega (Charbonnières‐les‐Bains, France).

PCR was performed with DreamTaq polymerase (Thermo Fischer Scientific, Courtaboeuf, France). Primers for SPINK2 and glyceraldehyde‐3‐phosphate dehydrogenase (GAPDH) were designed using Primer‐BLAST (http://www.ncbi.nlm.nih.gov/tools/primer‐blast/). Primers were 5′‐ATGGCGGCGAAGCTGACG‐3′ (sense) and 5‐TCAGCACATTCCACTC‐3′ (antisense) for SPINK2 (256 bp), and 5‐TGCTGCCCAGAACATCATCC‐3′ (sense) and 5′‐ATCAGCAGCAGCCTTCACTACC‐3′ (antisense) for GAPDH (194 bp). The reaction mixture for PCR contained 1 µl of complementary DNA, 2 µl of 10× green buffer, 0.5 µl of (10 µM) forward and reverse primers each, 0.4 µl of (10 mM) dNTP mix, 0.2 µl of (5 U) Dream Taq polymerase and 15.4 µl of nuclease‐free water. The thermal cycling conditions were 95°C for 2 min, followed by 30 cycles of 95°C for 30 s, 60°C for 30 s and 72°C for 20 s and a final elongation at 72°C for 10 min. Samples were then submitted to agarose electrophoresis.

### 2.5 In situ hybridization

Fragments of testis, epididymis, and ductus deferens were fixed in PAF 4%/phosphate‐buffered saline for 24 hr at 4°C, dehydrated, embedded in paraffin and serially sectioned (10 µm). Sections were deparaffinized in xylene, rehydrated gradually and washed in water. Sections were treated (deproteination, acetylation) and dehydrated again. Hybridization was performed at 65°C for 12 hr in the presence of 1 µg of antisense or sense probes. After posthybridization washes, digoxigenin (DIG) activity was detected using anti‐DIG‐AP antibody (12 hr at 4°C) and revealed with NBT‐BCIP. SPINK2 PCR fragment was subcloned in the pCS2 + vector. Sense and antisense probes were generated using DIG RNA labeling kit (LifeScience, Saint‐Quentin Fallavier, France).

### 2.6 Western blot (WB)

Keyhole limpet hemocyanin‐coupled SPINK2 peptide corresponding to residues 34–49 of SPINK2 was used to immunize rabbits and to produce a polyclonal affinity‐purified anti‐SPINK2 (GenScript, Piscataway, NJ). For anti‐SPINK2 antibody characterization, 40 µg of total protein from liver, testis, and epididymis and 20 µg of total protein from pool of sperm and seminal plasma were separated on 15% sodium dodecyl sulphate polyacrylamide gel (SDS‐PAGE) gels under reducing conditions, blotted onto nitrocellulose membranes, which were further blocked for 1 hr in 5% milk TBS‐Tween 20 buffer.

For quantification of SPINK2 in seminal plasma of contrasted fertility rate chicken, 10 µg of proteins of individual roosters were used. SPINK2 was detected by anti‐SPINK2 (1:2,000) followed by anti‐rabbit secondary antibody conjugated with Alexa Fluor® 680 (Thermo Fischer Scientific, Courtaboeuf, France). Total protein staining of the membrane with SyproRuby blot stain (Thermo Fischer Scientific, Courtaboeuf, France) prior blocking served as a loading control. Images were obtained by scanning on a Fusion FX (SyproRuby stained membranes) or a Li‐Cor Odyssey Infrared Imager (Immunoblots). All images were digitalized and analyzed by Image Studio Lite Software (Li‐Cor Biosciences, Lincoln). For each lane, band intensity values were normalized relatively to the respective overall protein staining (Figure S2).

### 2.7 Immunohistochemical detection of SPINK2 in chicken tissues

Ten microns sections were prepared as described above. To achieve antigen staining, the slides were immersed in citrate buffer (10 mM, pH 6.0) and heated in a microwave oven (6 min, 440 W). Nonspecific binding was prevented with TBS‐bovine serum albumin 0.1% buffer incubation before incubation overnight at 4°C in a humidified chamber in the presence of SPINK2 antibody (1:200). Subsequently, sections were incubated with anti‐rabbit‐AP antibody (1:200) for 1 hr. The staining was revealed using NBT/BCIP for one to 2 hr at 37°C. Negative controls included sections that were incubated without the primary antibody and with the secondary antibody only.

### 2.8 Immunofluorescence detection of SPINK2 in chicken sperm

Fresh spermatozoa were fixed for 5 min 4% PAF solution and labeled as previously described (Nguyen et al., 2014) with anti‐SPINK2 antibody (1:200) followed by anti‐rabbit secondary antibody conjugated with Alexa Fluor® 584 (Thermo Fisher Scientific, Courtaboeuf, France). Negative controls included sperm that were incubated without the primary antibody and with the secondary antibody only. Images obtained were analyzed and quantified using Fiji (Schindelin et al., 2012) to detect differences of labeling between roosters.

### 2.9 Purification of SPINK2 from chicken seminal plasma and identification by MALDI‐TOF mass spectrometry

SPINK2 protein was purified from seminal plasma by Reverse Phase HPLC with a HPLC Agilent 1100 series (Agilent technologies). Seminal plasma was submitted to ultracentrifugation (cut‐off 30 kDa) for 10 min at 4,000*g*. Eluate (proteins < 30 kDa) was collected and further submitted to chromatographic separations (X Bridge column C 18.5 µm [4.6 × 150 mm]). Gradient elution was performed using a mobile phase A consisting of 90:10 (v/v) acetonitrile: water with 0.1% trifluoroacetic acid (TFA), and a mobile phase B consisting of acetonitrile with 0.1% TFA. The gradient was initiated at 0% B upon injection and increased to 70% B using a linear gradient over 75 min. The flow rate was 0.5 ml/min. Collected fractions were evaporated (Speed Vac Savant Instrument, Bioblock Scientific, Illkirch, France) at room temperature and resuspended in water before analysis. Fractions were analyzed by WB using specific SPINK2 antibody and by MALDI‐TOF to confirm the purity of the SPINK2 containing sample.

Mass spectrometry analysis was performed as follows at the CIRE‐PAIB platform (UMR‐PRC, INRA, Nouzilly, France). For each fraction, 1 µl was spotted onto a Bruker 384‐position AnchorChip (Bruker Daltonics, Bremen, Germany) sample plate and overlayed with a α‐cyano‐4‐hydroxycinnamic acid saturated matrix solution freshly prepared by dissolving the solid crystals in 1 ml of 50% acetonitrile/50% H_2_O in presence of 0.1% TFA reagent and sonicated for 10 min before application. The sample and the matrix (1:1, v/v) were loaded on the target using the dried droplet method. Spectra were acquired using an UltrafleXtrem MALDI‐TOF/TOF instrument (Bruker) equipped with a Smartbeam laser at 2 kHz laser repetition rate that was controlled by FlexControl 3.0 software (Bruker Daltonics, Bremen, Germany). Spectra were obtained in positive linear ion mode in the *m*/*z* 1,000–30,000 range. Spectra were collected from each spot as a sum of 1,000 laser shots, in two shot steps (total of 2,000 spectra per spot). The accelerating voltage was set to 25 kV. External calibration was performed using mixture contained Glu1‐fibrinopeptide B, ACTH (fragment18–39), insulin, and ubiquitin, all at 1 pmol/µl, 2 pmol/µl cytochrome *c*, 4 pmol/µl myoglobin, and 8 pmol/µl trypsinogen. FlexAnalysis software version 3.4 (Bruker Daltonics) were applied for data processing.

### 2.10 Inhibition of the activity of target proteases by SPINK2 using gelatin zymography

Twenty micrograms of the F3 fraction eluted from benzamidine chromatography were incubated with increasing amounts (0–5 µM) of purified SPINK2. Samples were then separated on 10% SDS‐PAGE gels under nonreducing conditions, without boiling. After migration, gels were washed with a 2.5% solution of Triton 100× and incubated with serine protease activity buffer (Tris‐HCl 50 mM, NaCl 50 mM, pH 7.8) during 18 hr at 37°C. Gels were stained with Coomassie brilliant blue R‐250 solution for 30 min. Stained gels were washed in 40% methanol and 7% acetic acid solution. Areas of proteolysis appeared as clear zones on a blue background.

### 2.11 Purification and identification of serine proteases from sperm extracts

Pooled semen (7 ml) was centrifuged at 600*g* for 10 min at 20°C. The supernatant was discarded and sperm was centrifuged again at 12,000*g* for 10 min at 4°C. Sperm pellet was resuspended in 6 M urea, placed in an ice bath and sonicated for 15 s, set at 40% relative output and centrifuged at 14,000*g* for 15 min at 4°C. The supernatant was collected and fractionated by affinity chromatography on benzamidine sepharose 4 Fast Flow (Sigma Aldrich, St. Quentin Fallavier, France). Column was equilibrated with 10 volumes of washing buffer (50 mM Tris‐HCl, 0.5 M NaCl, pH 7.4). Sperm extract sample was loaded and unbound fraction was collected. The column was then washed with washing buffer until the DO 220 nm reached 0. Elution was performed with 50 mM glycine pH 3.0. Fractions (500 µl) were collected on Eppendorf tube containing Tris‐HCl 1 M pH 9.0 for pH neutralization. Protein concentration of each fraction was determined using absorbance at 280 nm and further analyzed by mass spectrometry for protein identification.

After in‐solution digestion, peptide mixtures were analyzed by on‐line nanoflow liquid chromatography tandem mass spectrometry (nanoLC‐MS/MS) at the CIRE‐PAIB platform (UMR‐PRC, INRA). All experiments were performed on a dual linear ion trap Fourier transform mass spectrometer LTQ Orbitrap Velos (Thermo Fisher Scientific, Bremen, Germany) coupled to an Ultimate® 3000 RSLC Ultra High Pressure Liquid Chromatographer (Dionex, Amsterdam, The Netherlands) controlled by Chromeleon Software (version 6.8 SR11; Dionex, Amsterdam, The Netherlands). Sample was desalted and concentrated for 10 min at 5 µl/min on an LCPackings trap column (Acclaim PepMap 100 C18, 75 µm inner diameter × 2 cm long, 3 µm particles, 100 Å pores). The peptide separation was conducted using a LCPackings nanocolumn (Acclaim PepMap C18, 75 µm inner diameter × 50 cm long, 2 µm particles, 100 Å pores) at 300nl/min. Column was equilibrated with 96% solvent A (0.1% formic acid, 97.9% water, 2% acetonitrile [v/v/v]) and 4% solvent B (0.1% formic acid, 15.9% water, 84% acetonitrile [v/v/v]). Then a linear gradient was started from 4% to 60% of solvent B for 90 min and finally a stage at 99% solvent B was applied for 10 min. Data were acquired in positive mode in data‐dependent mode to automatically switch between high resolution full‐scan MS spectra (R 60 000) collected in profile mode and low‐resolution CID‐MS/MS in centroid mode (*m*/*z* 300–1,800). The 20 most intense peptide ions with charge states ≥ 2 were sequentially isolated and fragmented in the high pressure linear ion trap by low energy CID (collision energy 35%, activation time 10 ms, Qz 0.25). Dynamic exclusion is activated during 30 s with a repeat count of 1. MS/MS ion searches were performed using Mascot search engine version 2.3.2 (Matrix Science, London, UK) via Proteome Discoverer 2.1 software (Thermo Fisher Scientific, Bremen, Germany) against NCBIprot_chordata. The search parameters included trypsin as a protease with two allowed missed cleavages and carbamidomethyl cysteine, methionine oxidation and acetylation of N‐term protein as variable modifications. The tolerance of the ions was set to 5 ppm for parent and 0.8 Da for fragment ion matches. Peptides and proteins identified by MASCOT were validated using “Peptid Prophet” and “Protein Prophet” algorithm with Scaffold software (version 4.8.4, Proteome Software, Portland, OR). Protein identifications were accepted if they contained at least two identified peptides.

### 2.12 Seminal plasma amidase activity and inhibition assay with purified SPINK2

Twenty micrograms of proteins from seminal plasma of roosters with contrasted fertility (*n* = 8 and 6 for layer and broiler lines, respectively) or 200 ng of proteins from affinity chromatography fraction were incubated in a 50 mM Tris‐HCl buffer, pH 8.0 at 37°C for 20 min. Trypsin and seminal plasma amidase activity were measured according to the method described by Geiger and Fritz (1983) with modifications described by Slowinska et al. (2012), using Na‐benzoyl‐d,l‐arginine 4‐nitroanilide hydrochloride (BAPNA; Sigma Aldrich) as substrate at a final concentration of 600 µM and by measuring the absorbance at 410 nm at 37°C during 20 min. Each reaction was performed in triplicate using a microplate reader (Tecan, Infinite 200, Tecan France SAS, Lyon, France). For inhibition assays, 20 µg of proteins extracted from sperm or 200 ng of proteins from affinity chromatography fraction were incubated with increasing amounts (0–800 nM) of purified SPINK2 or α1‐antitrypsin (MP Biomedicals, Illkirch, France) in a 50 mM Tris‐HCl buffer, pH 8.0 at 37°C for 20 min. The remaining activity of serine proteases was evaluated by addition of BAPNA, as described above.

### 2.13 Statistical analysis

Data are represented as means ± SEM. Statistical analysis for multiple comparisons was performed using nonparametric Kruskal–Wallis test follow by Tukey's least significant difference (LSD). Percentages were transformed to arcsine square‐root before analysis. The data were analyzed using R (http://cran.r‐project.org; R version 3.5.1). The level of significance was set at a *p* < 0.05.

## 3 RESULTS

### 3.1 Characterization of SPINK2 sequence

In our previous proteomic investigation (Labas et al., 2015), ClTI‐1 protein was identified as a 55 amino‐acid proteoform with several posttranslational modifications including a proteolytic event leading to the excision of the N‐terminal methionine, and three disulfide bridges. Using a protein and genomic sequence alignment tool, we found that this proteoform matched with 100% of sequence coverage and an *E*‐value at 3.0 E‐36, with the *Gallus gallus* SPINK2 protein (predicted serine protease inhibitor Kazal‐type 2, isoform X2). The whole sequence of 81 amino acids (aa), corresponding to Gene ID 770729, was composed of a signal peptide (aa 1–26) and a Kazal domain (aa 27–81) (Figure S1A), Arg^42^–Asp^43^ residues representing the P1‐P’1 reactive site. Comparison of predicted SPINK2/ClTI‐1 protein sequences of *G. gallus* (Gg), *Meleagris gallopavo* (Mg), *Coturnix japonica* (Cj), *Danio rerio* (Dr), *Xenopus tropicalis* (Xt), *Equus caballus* (Eq), *Homo sapiens* (Hs), *Bos taurus* (Bt), *Sus scrofa* (Ss), *Mus musculus* (Mm) revealed a significant similarity in the Kazal domain (Figure S1B) with conservation of the six characterized cysteines.

### 3.2 Expression profile of SPINK2 transcript and protein in chicken male

**Figure 1:**
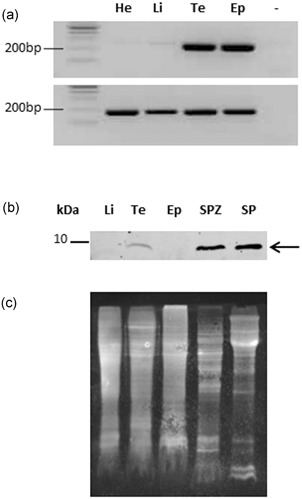
SPINK2 expression profile in chicken tissues. (a) SPINK2 messenger RNA (top panel) was investigated by RT‐PCR (see specific primers in Section 2), in heart (He), liver (Li), testis (Te), epididymis (Ep); no PCR substrate as negative control (−). RT‐PCR for glyceraldehyde‐3‐phosphate dehydrogenase (bottom panel) is shown as a housekeeping gene. (b) Western blot using anti‐SPINK2 antibody with protein extracts from liver (Li), testis (Te), epididymis (Ep), spermatozoa (SPZ), and seminal plasma (SP). Molecular weight is indicated on the left. The arrow indicates SPINK2 signal. (c) Membrane stained with SyproRuby to evaluate the protein loading. RT‐PCR: reverse transcription polymerase chain reaction

**Figure 2:**
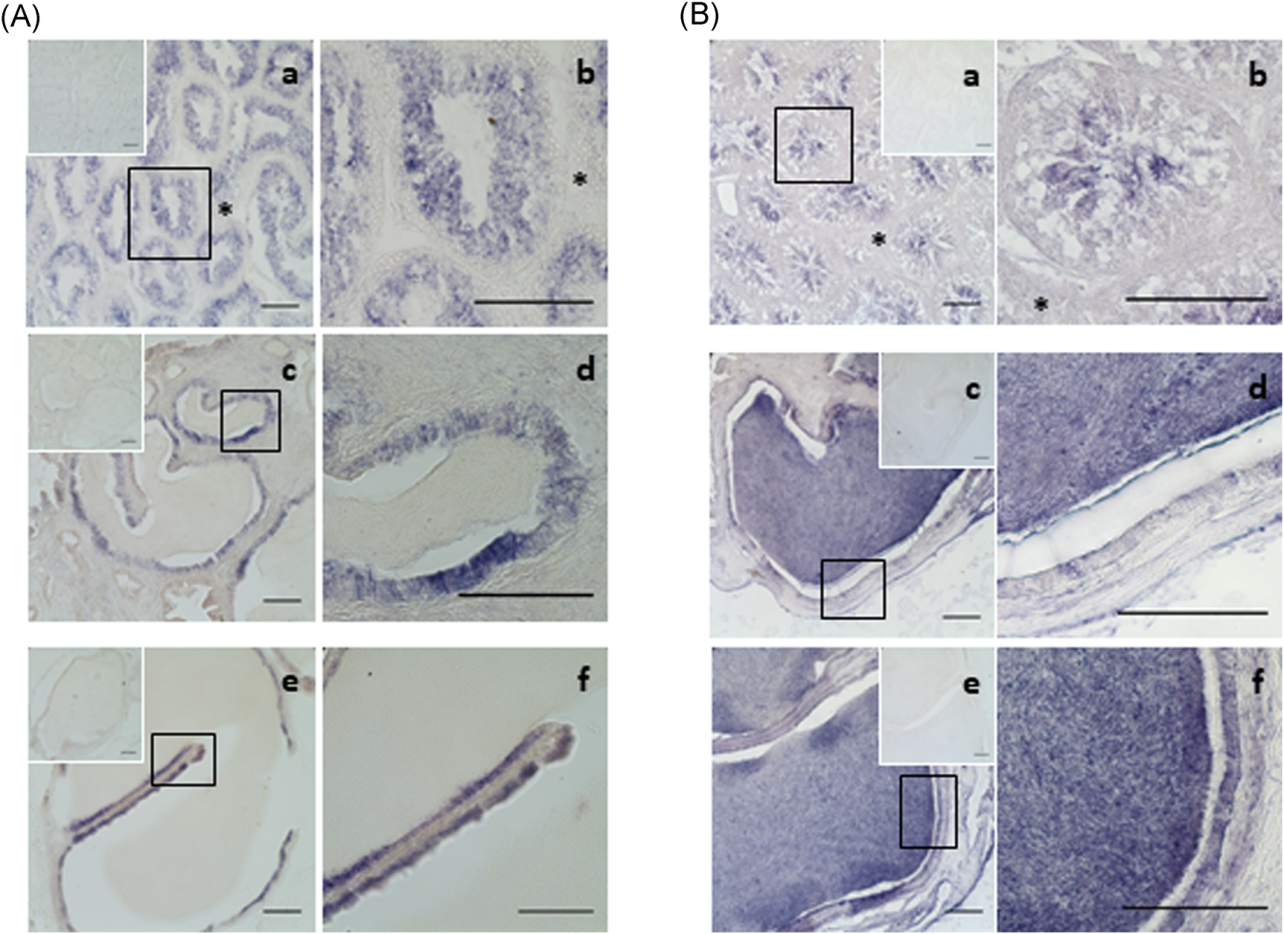
SPINK2 transcripts and protein in the chicken reproductive tissues. (A) Localization of SPINK2 transcripts by in situ hybridization. Photomicrographs of testis sections (a,b), epididymis sections (c,d), and ductus deferens sections (e,f) hybridized with antisense SPINK2 riboprobe. No signal was observed when sense riboprobe (as negative control) is hybridized (upper insets). Interstitium is indicated by an asterisk. Bars = 100 µm. (b) Immunohistochemical localization of SPINK2 protein. SPINK2 is revealed in the chicken testis (a,b), epididymis (c,d), and ductus deferens (e,f) by a purple staining. Interstitium is indicated by an asterisk. No signal was observed when primary antibody is omitted (upper insets). Bars = 100 µm

By RT‐PCR using specific primers, we observed that the signal was much stronger in the testis and epididymis in contrast to the liver and heart where SPINK2 mRNA was barely detectable (Figure 1a). Using in situ hybridization, we detected SPINK2 transcripts in the whole epithelium of seminiferous tubules of testis, but neither in the interstitium, nor in the spermatozoa inside the lumen (Figure 2A‐a,b). *SPINK2* mRNA was also detected in epididymal and ductus deferens epithelium (Figure 2A‐c–f). No signal was detected in the lumen.

By WB, we detected an expected 5–10 kDa band in chicken seminal plasma, sperm extracts, and testis (Figure 1b). In the testis and the epididymis, the protein was detected in the lumen with a strong signal (Figure 2B), suggesting that the protein was secreted from the epithelium into the lumen and accumulated in the seminal plasma where it may absorbed at the sperm surface (Figure 1b).

**Figure 3:**
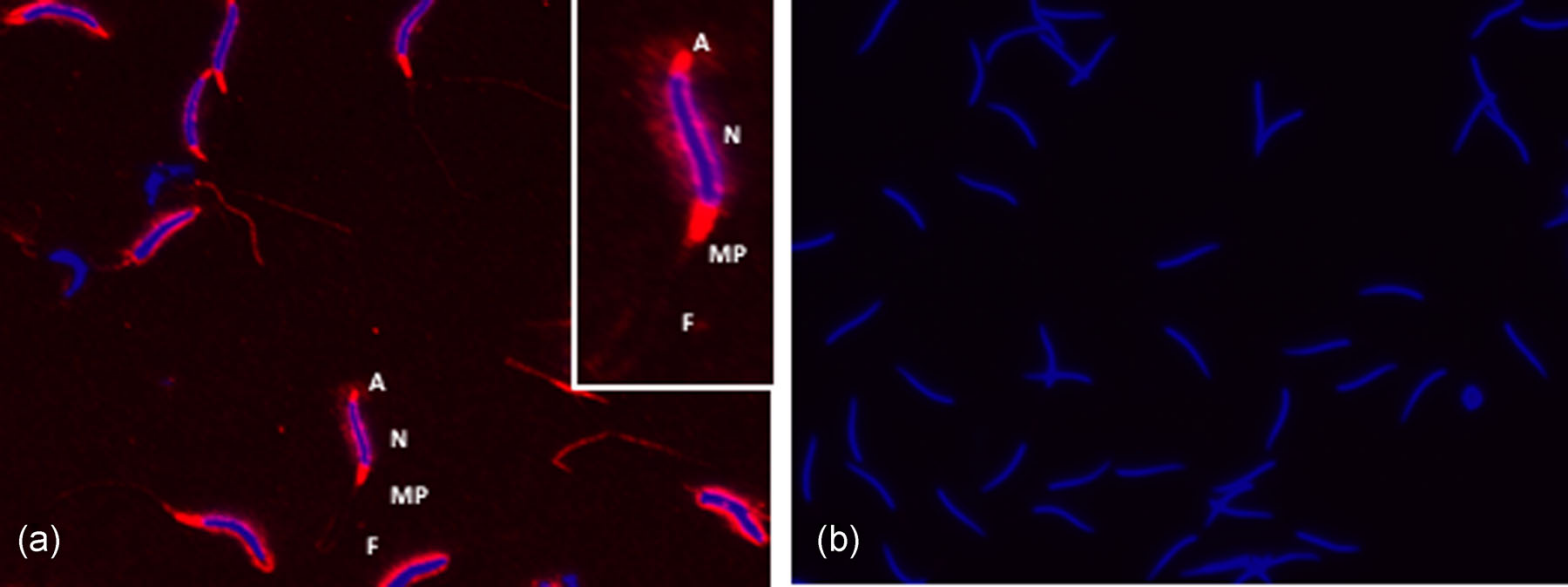
Immunofluorescence of SPINK2 in chicken sperm. (a) Expression of SPINK2 on the sperm. (b) No signal was detected in the control slide (incubated with the secondary antibody only). Red color: SPINK2 localization in acrosome (a), midpiece region (MP) and flagella (F); blue color: staining of the nucleus (N)

By immunofluorescence, SPINK2 was localized in the acrosome and midpiece region of ejaculated spermatozoa (Figure 3a). No signal was detected in the sperm incubated without primary antibody and with the secondary antibody only (Figure 3b).

### 3.3 Inhibitory activity of SPINK2 protein purified from chicken seminal plasma

We collected three fractions (Figure S3A) from reverse phase chromatography (final step of purification, see Section 2) that were further analyzed by MALDI‐TOF mass spectrometry (data not shown) and WB (Figure S3B) to identify fractions containing SPINK2 protein. WB revealed a single band in the second fraction (F2) and MALDI‐TOF spectra confirmed that SPINK2 was present in the F2 fraction at *m*/*z* 3,013 and 6,025 ± 0.05% corresponding to di‐ and mono‐charged forms, respectively.

**Figure 4:**
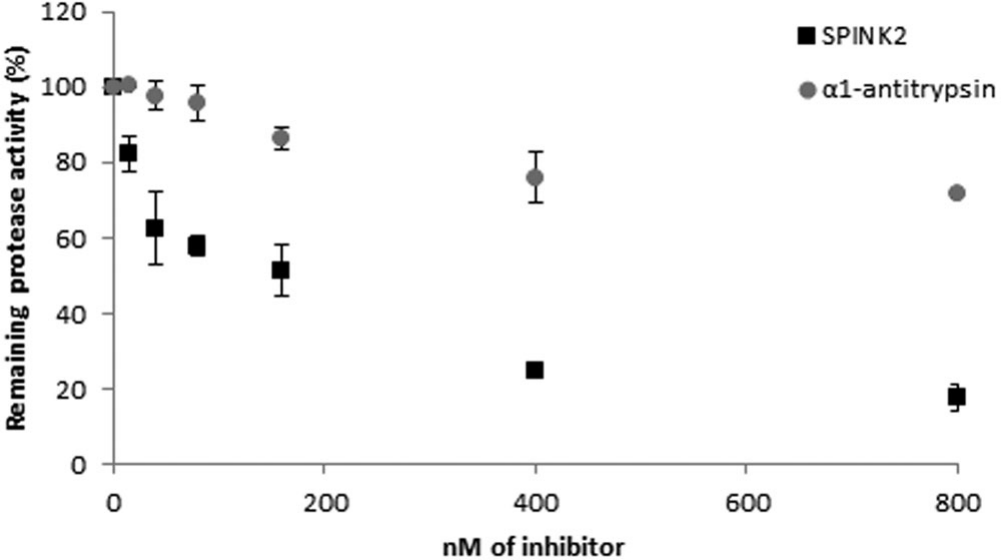
Effect of SPINK2 and α1‐antitrypsin on serine protease activity in sperm extracts. Twenty micrograms of proteins extracted from sperm were incubated with increasing amounts (0–800 nM) of purified SPINK2 (■) and α1‐antitrypsin (○) in a 50 mM Tris‐HCl buffer, pH 8.0 at 37°C for 20 min. The percentage of remaining sperm protease activity is indicated in *y*‐axis and inhibitor concentration (nM) is indicated in *x*‐axis

The inhibitory activity of purified SPINK2 was first assessed against trypsin to verify that the purification process did not affect SPINK2 inhibitory activity. One hundred nanomolar of SPINK2 inhibited 63% (2 nM) of trypsin activity (data not shown). The inhibitory activity of SPINK2 was further assessed on sperm extracts. SPINK2 induced a dose‐dependent inhibition of spermatozoa proteases with trypsin/trypsin‐like activities: 75.3% and 82.6% of inhibition with 400 and 800 nM of purified SPINK2, respectively (Figure 4, ■). As a control, we used the α1‐antitrypsin inhibitor, known to partially inhibit acrosin (Hermans, Monard, Jones, & Stone, 1995), at the same concentration than SPINK2 and as expected, we showed a weak inhibition (29%) in sperm extracts (Figure 4, ●).

### 3.4 Physiological target proteases of SPINK2 protein

**Figure 5:**
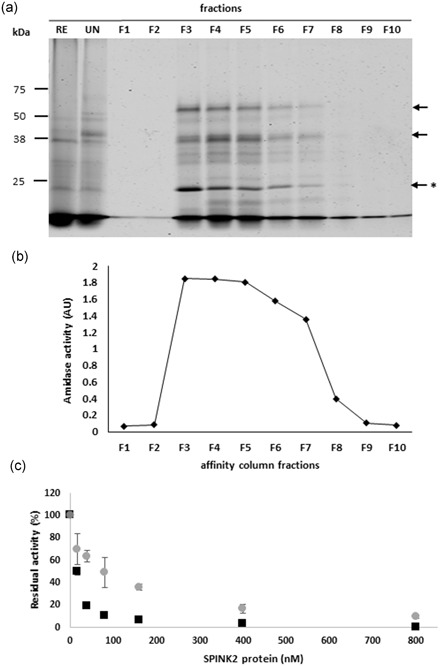
Analysis of sperm extracts after affinity chromatography (benzamidine sepharose). (a) Electrophoretic profiles of fractions collected after affinity chromatography on sperm extracts. Black arrows indicate the more abundant proteins, * indicates the active form of the acrosin. (b) Amidase activities. Two hundred nanograms of proteins from each fraction were incubated in presence of BAPNA in a 50 mM Tris‐HCl buffer, pH 8.0 at 37°C for 20 min. AU, absorbance unit at 410 nm. (c) Effect of SPINK2 on serine protease activity of F3 fraction (■) and F4 fraction (○). Two hundred nanograms of proteins from F3–F4 fractions were incubated with increasing amounts (0–800 nM) of purified SPINK2 in a 50 mM Tris‐HCl buffer, pH 8.0 at 37°C for 20 min. The percentage of remaining sperm protease activity is indicated in *y*‐axis and inhibitor concentration (nM) is indicated in *x*‐axis. RE: row extract; UN: unbound fraction; F1‐F10: eluted fractions

**Figure 6:**
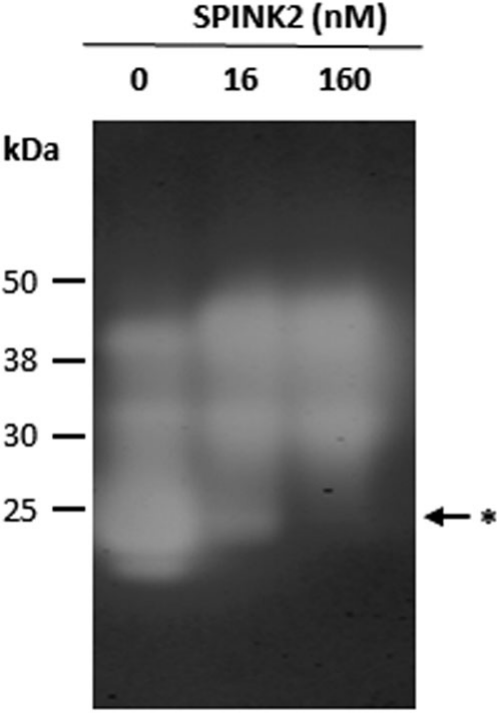
Effect of SPINK2 on serine proteases activity in F3 fraction eluted from benzamidine chromatography by reverse zymography. Gelatin zymograms of F3 fraction incubated with or without SPINK2 inhibitor reveal serine protease activity. Three major bands of proteolysis were identified at 38, 30, and 25 kDa corresponding to precursor and active forms of acrosin, respectively. The asterisk indicates areas where SPINK2 inhibits serine proteases activity. Lanes: 0, F3 fraction without SPINK2; 16, F3 fraction + 16 nM of SPINK2; 160, F3 fraction + 160 nM of SPINK2

We first performed a benzamidine chromatography to isolate serine proteases composing the sperm extract (Lessley & Brown, 1978; Thurston, Korn, Froman, & Bodine, 1993). Results are displayed in Figure 5a. We observed a complex protein content with more abundant proteins around 50, 38, and 25 kDa (black arrows). We measured the amidase activity in each fraction and we showed that the activity was very high from fractions F3 to F5 and then decreased progressively (Figure 5b), which is correlated positively with the abundance protein profile (Figure 5a). Fractions F1–F2 and F9–10, as well as unbound fraction (data not shown), lacked any activity. F3 and F4 fractions, where the amidase activity was higher, were submitted to inhibition assay with SPINK2. We showed that SPINK2 induced a dose‐dependent inhibition of proteases contained in the fraction: 99.6% and 90.4% of inhibition with 800 nM of purified SPINK2, for F3 and F4 fractions, respectively (Figure 5c). Fractions were further analyzed by nanoLC‐MS/MS. We identified 27 distinct proteins including only two proteases (Table 1). The two proteases are serine proteases and correspond to two acrosin isoforms: ACR, Gene ID 426864, 39 kDa and ACRL, Gene ID 769176, 38 kDa. The presence of 25 additional proteins in the fraction retained by benzamidine chromatography, may reflect some nonspecific binding and/or protein interaction with acrosin proteins. We further performed reverse zymography on F3 fraction obtained after affinity chromatography of a sperm extract sample. We incubated F3 fraction, containing acrosin, with increasing concentration of SPINK2 protein 0–160 nM) and we measured the remaining activity of serine proteases by zymography. Without inhibitor, gelatin‐degrading proteases were detected in F3 fraction (Figure 6, line 0). Three major bands of proteolysis were identified at 38, 30, and 25 kDa corresponding to latent (precursor) and active form of acrosin, respectively (Figure 6, line 0, white bands). It is noteworthy that zymography allows to detect the activity of both latent and active forms of proteases, as published previously (Toth, Sohail, & Fridman, 2012). The serine protease activity corresponding to a 25 kDa band, was completely inhibited in a dose‐dependent manner by SPINK2 (Figure 6, lines 16 and 160, asterisk). The corresponding 25 kDa band was further analyzed by mass spectrometry for protein identification. Data showed that this 25 kDa band contains both acrosin and acrosin like proteins, as the unique serine proteases found in this sample (Figure 5a, asterisk). The analysis of mass spectrometry results also suggests that acrosin and acrosin‐like are in their active form: no peptides matching with propeptide sequence could be identified on the 16 and 38 peptides identified for acrosin and acrosin‐like, respectively.

**Table 1:** Proteins identified by mass spectrometry in fractions after benzamidine chromatography with sperm

Description (*Gallus gallus)*	Gene symbol	Gene ID	Percent coverage (%)	MW (kDa)	Normalized emPAI
Dihydropyrimidinase	DPYS	420266	30.1	58	1.39
**Acrosin‐a**	**ACR**	**426864**	**18.9**	**39 **	**1.08**
**Acrosin‐b**	**ACR**	**769176**	**15.7**	**38 **	**0.79**
Sperm autoantigenic protein 17	SPA17	768714	17.4	16	0.47
1‐Phosphatidylinositol 4,5‐bisphosphate phosphodiesterase zeta‐1	PLCZ1	418182	18.1	73	0.36
Peptidylprolyl isomerase like 6	PPIL6	421764	19.0	33	0.33
Proteasome C1 subunit, partial	PSMB5	396003	13.7	22	0.33
Calcium binding tyrosine phosphorylation regulate	CABYR	107052002	13.6	35	0.31
Hexokinase 3	HK3	768421	7.43	110	0.26
Cluster of serum albumin precursor	ALB	396197	12.8	70	0.24
Neural EGF like 2	NELL2	417799	8.46	91	0.24
NME/NM23 family member 8	NME8	428461	6.74	66	0.16
PIT54 protein	PIT54	395364	7.45	51	0.13
Dynein axonemal intermediate chain 1	DNAI1	431654	5.82	81	0.13
Tektin‐5	TEKT5	416635	5.18	56	0.12
MYCBPAP associated protein	MYCBPAP	422101	4.07	109	0.092
Acyl‐CoA synthetase bubblegum family member 2	ACSBG2	420090	3.68	81	0.082
Adenylate kinase 7	AK7	428907	4.62	82	0.081
Dynein axonemal heavy chain 7	DNAH7	424048	3.77	459	0.073
Dynein axonemal heavy chain 3	DNAH3	427004	2.91	463	0.072
Cilia‐ and flagella‐associated protein 69 isoform X1	CFAP69	420542	2.68	105	0.063
Importin‐5	IPO5	418783	2.37	124	0.053
Dynein axonemal intermediate chain 10	DNAH10	416818	2.45	528	0.050
Androglobin	ADGB	421618	1.75	182	0.036
Cilia‐ and flagella‐associated protein 43 isoform X1	CFAP43	423879	2.24	194	0.034
Dynein axonemal intermediate chain 12	DNAH12	416004	1.46	453	0.029
Vacuolar protein sorting‐associated protein 13 C isoform X1	VPS13C	415376	0.56	422	0.015

*Note*. Proteases are shaded in gray.

To conclude, acrosin and acrosin‐like proteins are (a) the only proteases isolated by the affinity chromatography with benzamidine, (b) displayed a high inhibition rate with SPINK2, and (3) are on the top list of the proteins identified by mass spectrometry (normalized EmPAI = 1.08 and 0.79; Table 1). Altogether, these results indicate that chicken acrosin proteins are the physiological targets for SPINK2. The peptides obtained by proteomic analysis enabled to clearly identify two forms of acrosin: acrosin (18.9% recovery rate) and acrosin‐like (15.7% recovery rate, EmPAI = 0.79, 38 kDa, Table 1; Figures 5 and 6), which share 68% protein sequence identity.

### 3.5 Correlations between seminal SPINK2 protein amount, amidase activity, and male fertility

**Figure 7:**
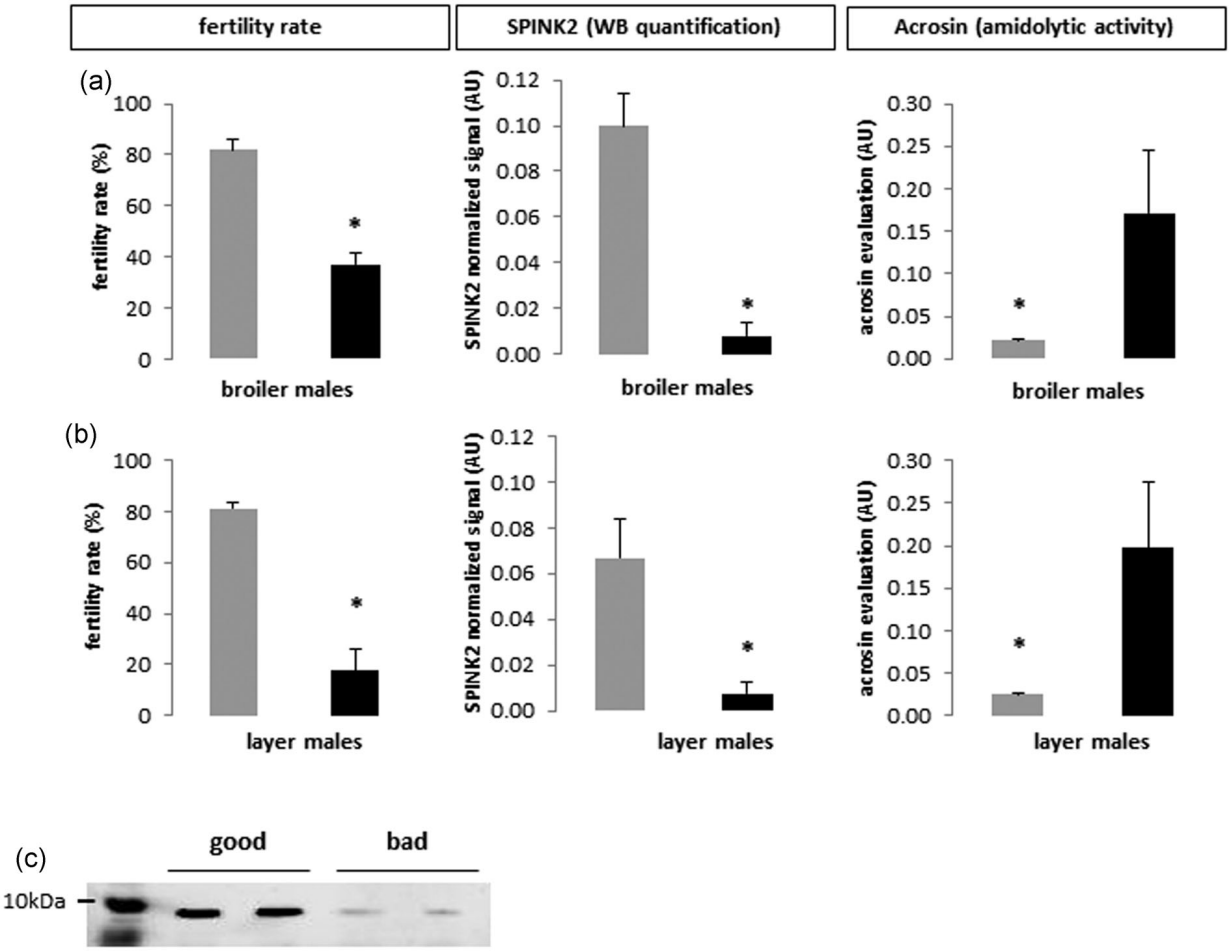
SPINK2 WB quantification, acrosin amidolytic activity and fertility rate in different chicken lines. (a,b) SPINK2 amount WB quantification, acrosin evaluation amidolytic activity, and fertility rate of good (gray histograms) and bad (black histograms) male breeders from broiler (a) and layer (b) lines. Values represent means ± SEM. Asterisks indicate significant differences between good and bad males (*p* < 0.05). (c) Western blot profiles of SPINK2 in seminal plasma of broiler breeder males (meat line) with contrasted fertility (high = good; low = bad). The loading control (SyproRuby counterstained membrane) is presented in Figure S2. WB: Western blot

**Figure 8:**
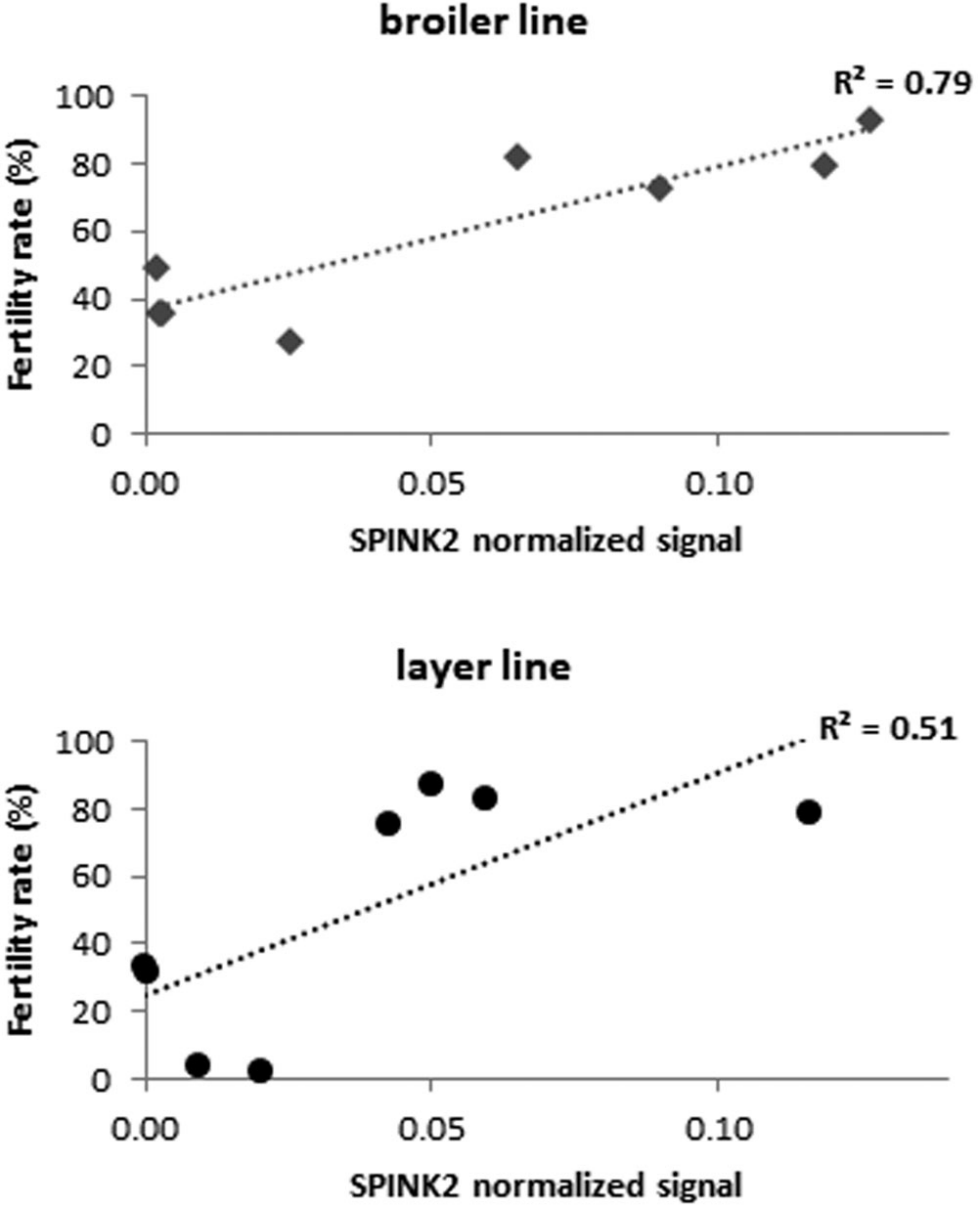
Correlation between SPINK2 protein amount and fertility rate. Positive correlations for males from three genetic lines (■ broilers and ● layers) between fertility rate and SPINK2 relative abundance

In our previous study (Labas et al., 2015), we identified ClTI‐1/SPINK2 as over abundant in seminal plasma of fertile roosters (free range chicken line). To further explore the physiological role of SPINK2, we measured the fertility of males from different genetic lines (broiler and layer strains). We selected male chickens with equivalent sperm concentration/ejaculate but with contrasted fertility (high and low) in each line. The mean male fertility capacities was specific to each line and high and low fertility rates differed between lines: for broiler breeder males (meat type), 81.85% and 37.18%, respectively (Figure 7a; *n* = 8) and for layer line males, 81.13% and 17.78%, respectively (Figure 7b; *n* = 8). We then quantified SPINK2 protein in seminal plasma of each characterized males group by WB (Figure 5c; individual broiler males). SPINK2 was detected in all samples but is significantly (*p* < 0.05) more abundant in seminal plasma from chickens with high fertility inside each line (Figure 7a,b; SPINK2 protein). The *R*² coefficients between the SPINK2 level in seminal plasma and the male fertility rate were 0.79 and 0.51, for broiler and layer lines, respectively (Figure 8). The same quantification was made for SPINK2 signal in sperm. SPINK2 was detected in all samples but at a lowest amount than in the seminal plasma. The level of protein is similar in sperms from chickens with high or low fertility (*p* = 0.258); data not shown).

In parallel, we evaluated the acrosin amidolytic activity in seminal plasma of broiler and layer males (Figure 7a,b; acrosin amidolytic activity) and we compared with the SPINK2 protein amount (Figure 7a,b; SPINK2 WB quantification). We observed that the acrosin activity is lower in highly fertile males where the SPINK2 protein amount is high. This negative correlation corroborates the above data and strengthens the conclusion that acrosin is a SPINK2 target in the seminal plasma.

## 4 DISCUSSION

In the present study, we characterized ClTI‐1, one of the seminal plasma proteins identified in our previous study as potential markers of chicken fertility (Labas et al., 2015) and investigated its postgonadic function for the first time.

ClTI‐1/SPINK2 is a serine protease inhibitor that was previously identified in chicken liver and then found in chicken seminal plasma and sperm (Kubiak et al., 2009; Labas et al., 2015; Lessley & Brown, 1978). Sequences of ClTI‐1 were also referenced in protein databases of other avian species including turkeys, quails, doves, and ducks and of other non‐bird species such as mouse, zebrafish, *Xenopus*, and *Drosophila*. Most of these protein sequences were predicted consequently to automatic chicken genome annotation in databanks.

Previous in vitro studies showed that Kazal‐type protease inhibitors possess trypsin inhibition properties (Lee et al., 2011; Lin et al., 2008; Slowinska et al., 2014; Zalazar et al., 2012). In our study, we showed for the first time using sperm of physiologically normal and healthy animals, that SPINK2 displays trypsin and trypsin‐like inhibition activity on sperm proteases. High inhibition efficiency of SPINK2 suggested that physiological function of SPINK2 is associated with semen protection against proteolytic activities of trypsin‐like protease(s). Using benzamidine chromatography combined to amidase activity measurement, inhibition assay, and mass spectrometry analysis, we identified only two proteases, acrosin and acrosin‐like protein.

Using this benzamidine affinity chromatography, we were able to purify 27 proteins from seminal plasma but only two are proteases (acrosin and acrosin‐like; Table 1, in gray). The fact that 25 other proteins that are not proteases, are able to bind benzamidine may reflect some nonspecific interactions with the matrix beads or they might interact with acrosin/acrosin‐like proteases. Most of these nonprotease proteins have low EmPAI values (Table 1), which reflect low abundance and corroborate the above sentence. Nevertheless, we showed by these data that acrosin from seminal plasma binds benzamidine serine protease inhibitor as expected (thus they are both active in seminal plasma) and that the only serine proteases present in seminal plasma are acrosin and acrosin‐like. Moreover, this fraction containing only acrosin and acrosin‐like as serine proteases was efficiently inhibited by SPINK2 (Figure 3; the hydrolytic potential of serine proteases contained in this fraction [thus acrosin and acrosin‐like] is inhibited in a dose‐dependent manner by SPINK2). In parallel, the zymography performed with purified SPINK2, showed the disappearance of some bands, which after proteomic analyses were shown to contain acrosin and acrosin‐like. Among the proteins identified in these bands, acrosin and acrosin‐like are the only serine proteases thus the only protease targets for SPINK2 inhibition. Thus, these two latter experiments demonstrate that acrosin and acrosin‐like are active in seminal plasma and are physiological targets of SPINK2. Acrosin, a trypsin like enzyme, is the most abundant protein identified in purified sperm extract fraction and the molecular weight of acrosin and acrosin‐like protein, 38 and 39 kDa, correspond to a major band identified on the SDS‐PAGE. Acrosin proteolytic activities are completely inhibited in the presence of small amount of seminal SPINK2. We thus infer that this protease is the physiological target of SPINK2. Acrosin is synthesized as proacrosin, a zymogen form, stored in the sperm acrosome and expected to be activated in a mature form at the time of fertilization, when acrosome enzymes are released in the oocyte environment (Baba, Michikawa, Kashiwabara, & Arai, 1989). Acrosin activity is known to be regulated by Kazal inhibitors (Laskowski & Kato, 1980; Slowinska et al., 2010). In turkeys, two forms of acrosin have been described, acrosin and acrosin‐like (Slowinska & Ciereszko, 2012; Slowinska et al., 2010). In the present study, we identified for the first time two homologs of acrosin in the chicken species (38 and 39 kDa as proprotein forms of acrosin and 27 kDa as active protease). Both acrosin (GeneID 426864) and acrosin‐like (GeneID 769176) proteins are localized within a 13 kb locus on chromosome 1 in *G. gallus* genome. We found the turkey acrosin‐like specific peptide (SLQEYVEPYRVLQEAKVQLIDL) in the chicken acrosin sequence. Recently by coexpression of acrosin and SPINK2 proteins in human cell line HEK293, it was demonstrated that SPINK2 prevents cell proliferation arrest induced by proacrosin (Kherraf et al., 2017). We thus conclude that, in the chicken, like in the mouse, SPINK2 regulates the activity of acrosin to prevent uncontrolled/deleterious sperm proteolysis and too early acrosome reactions. However, it is necessary to highlight that acrosin from mammalian and avian species may have diverging functions since the sequence identity between these species varies from 36% to 43%, and since most avian acrosins lack the conventional proline‐rich propeptide that is required for activation of mammalian acrosins (Figure S4).

The chicken is known to be a very robust model to study candidate fertility marker proteins since the fertility evaluation is noninvasive and highly powerful in this species (availability of males with contrasted fertility) allowing us to complete the functional study by fertility tests (Soler et al., 2016). We showed positive correlations between fertility and SPINK2 amount in the seminal plasma of highly different genetic lines (meat and egg lines), confirming that SPINK2 function is important at the physiological level, regardless of the genetic background. We also revealed that the amidase activity (reflecting enzymatic activity of acrosin) is conversely correlated to the fertility rate and the SPINK2 amount. Our results demonstrated that SPINK2 amount in sperm should be a good candidate marker to predict male fertility.

Interestingly, in our previous proteomic study, acrosin, the main target of SPINK2 in sperm (as demonstrated in the present study), was also detected as differentially expressed in Label Rouge roosters’ semen with contrasted fertility (overexpressed in males with low fertility rate; Labas et al., 2015).

In mouse, *Spink2* genomic invalidation demonstrated the importance of the protein at the gonadic level. SPINK2 allowed spermatid differentiation and acrosome formation during spermatogenesis and was still present on the acrosome of mature sperm of mouse and human to protect it from proteolysis (Kherraf et al., 2017). In the present study, we demonstrated a role of SPINK2 at the postgonadic level. We showed that ClTI‐1/SPINK2 is preferentially expressed in the male reproductive tract (at both the mRNA and protein levels; Figures 1 and 2) but not in the liver and the heart, and is recovered in the seminal plasma where it interacts with sperm proteins. Our results suggest that, in avian species, diffuse secretory cells of the testis, efferent ducts, epididymis, and deferent ducts secrete SPINK2 allowing its accumulation in the seminal plasma. SPINK2 was previously suspected to be a secreted protein of mammals’ male tract since it was found in mice testis, epididymal, and seminal vesicles fluids. Bird sperm develop adaptations to avoid too early acrosome reactions. Consequently, they need very high antiprotease protections before reaching the site of fertilization.

To conclude, the ClTI‐1/SPINK2 protein, is a single domain Kazal‐type serine protease inhibitor, that targets the chicken acrosin and acrosin‐like proteins in seminal plasma. The role of seminal SPINK2 acrosin inhibitor in fertility is likely to prevent too early acrosin activation that would be deleterious for sperm cells. Seminal SPINK2 is present in higher amount in highly fertile males in contrast to acrosin that is over‐abundant in low fertile males. From these results, we strongly suggest that high seminal SPINK2 induce efficient inhibition of acrosin activity which is important for fertility. Thus, SPINK2 seems to be a relevant candidate fertility marker in chicken species. Whether SPINK2 has the same function and is a fertility marker also in other species is a crucial question that should be addressed in the future.

## CONFLICT OF INTERESTS

The authors declare that there are no conflict of interests.

## AUTHOR CONTRIBUTIONS

EB supervised the project. AT designed and performed experiments. SRG brought her expertize in proteases and inhibitors. JCP handled HPLC protein purification. SF and MG contributed to data analyses. AT wrote the manuscript with editorial assistance from EB, SRG and SF. All authors read and approved the final manuscript.

## Supporting information

Supporting information

Supporting information

Supporting information

Supporting information

Supporting information
