# Supplementary figures and images for: The seminal acrosin‐inhibitor ClTI1/SPINK2 is a fertility‐associated marker in the chicken
Source: Mol Reprod Dev. 2019 Apr 29;86(7):762–75. doi: 10.1002/mrd.23153 (PMC6767445; doi:10.1002/mrd.23153)

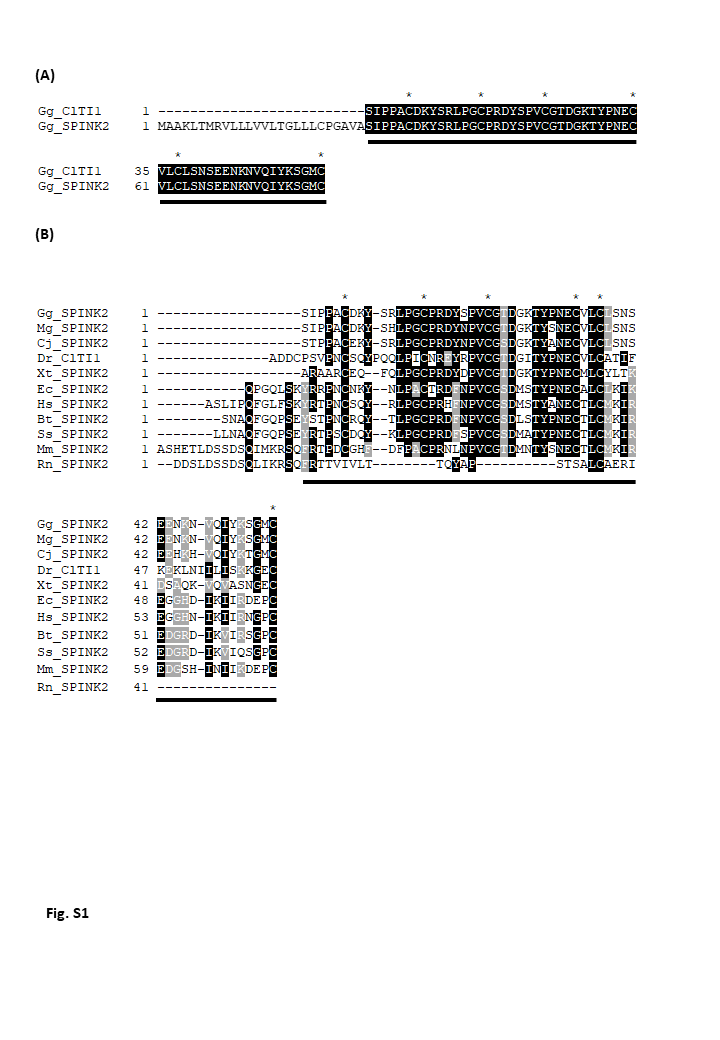

Supplement: Supplementary file 1 — Supporting information [file MRD-86-762-s001.TIF]

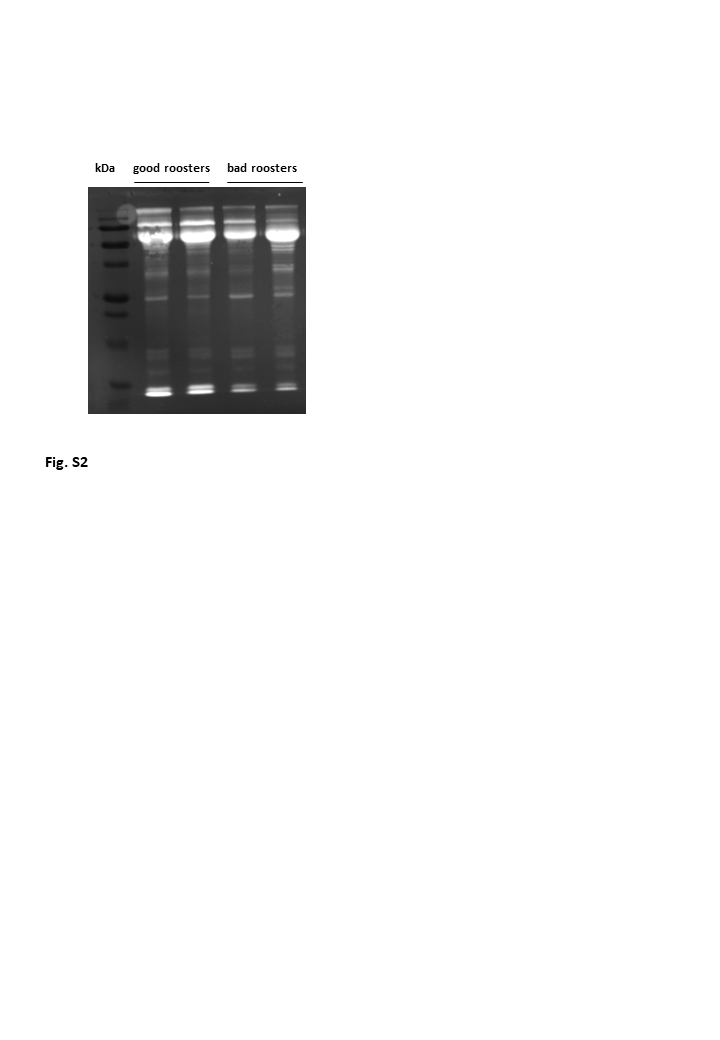

Supplement: Supplementary file 2 — Supporting information [file MRD-86-762-s002.TIF]

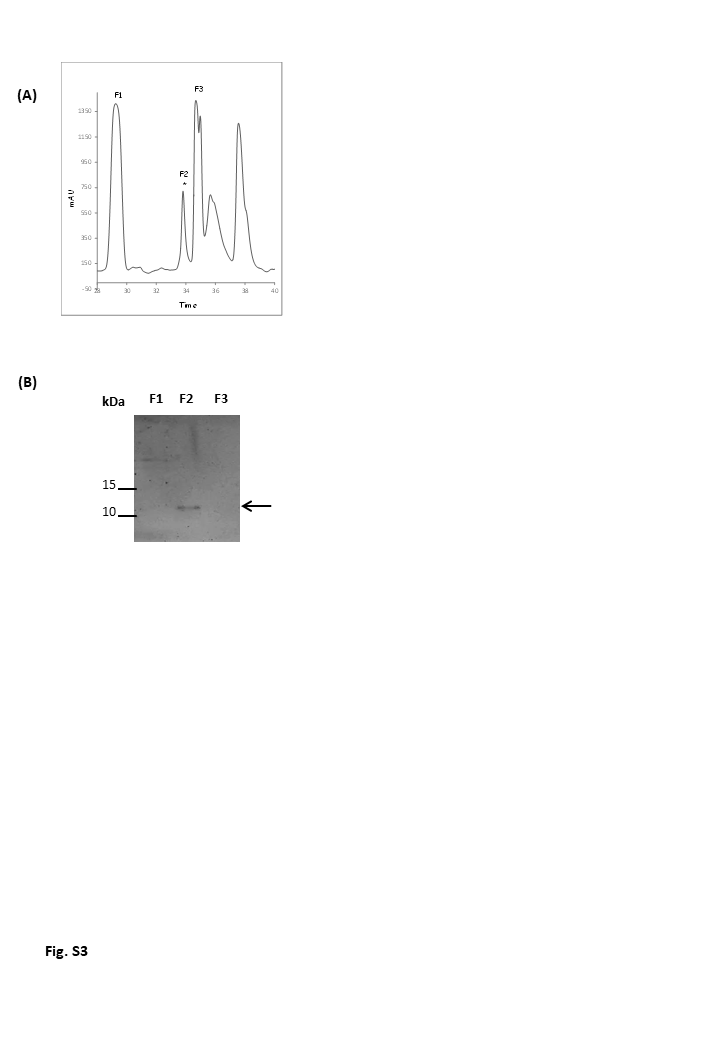

Supplement: Supplementary file 3 — Supporting information [file MRD-86-762-s003.TIF]

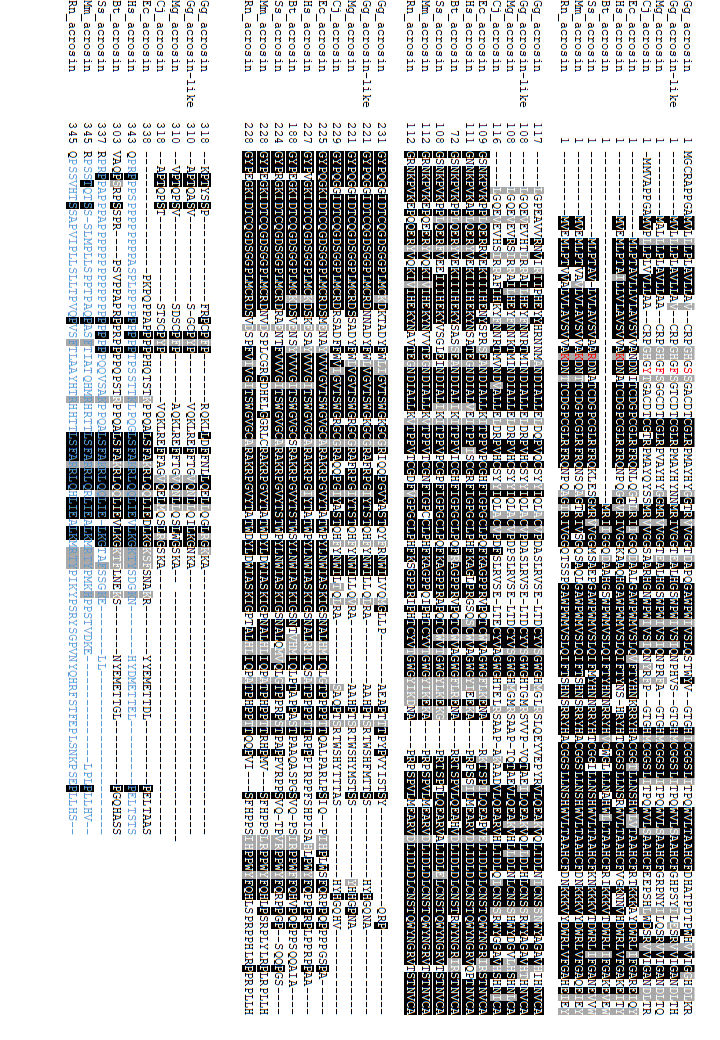

Supplement: Supplementary file 4 — Supporting information [file MRD-86-762-s004.TIF]
