# Supplementary material for: The seminal acrosin‐inhibitor ClTI1/SPINK2 is a fertility‐associated marker in the chicken
Source: Mol Reprod Dev. 2019 Apr 29;86(7):762–75. doi: 10.1002/mrd.23153 (PMC6767445; doi:10.1002/mrd.23153)
Supplement: Supplementary file 5 — Supporting information [file MRD-86-762-s005.docx]

**SUPPLEMENTARY MATERIAL**

**Figure S1: SPINK2 protein sequence conservation.** KAZAL domain is underlined, conserved Kazal domain cysteine are annotated with an asterisk. (A) Alignment of chicken ClTI-1 and SPINK2 protein sequences. (B) Alignment of putative SPINK2/ClTI-1 orthologues sequences in turkey (Mg), quail (Cj), zefrafish (Dr), Xenopus (Xt), horse (Ec), human (Hs), bovine (Bt), boar (Ss), mouse (Mm) and rat (Rn). Identical and similar amino-acids conserved between species are indicated in black and grey, respectively. Protein sequences alignments were performed using Clustal Omega (http://www.ebi.ac.uk/Tools/msa/clustalo/) and amino acid identity/similarity were highlighted using BoxShade (http://embnet.vital-it.ch/software/BOX_form.html).

**Figure S2: SyproRuby staining of nitrocellulose membrane of Western blot.** Membranes were stained with SyproRuby to evaluate the protein loading.

**Figure S3: Purification of SPINK2 from seminal plasma and MALDI-TOF identification.** (A) Profile of HPLC reverse phase purification. F1, F2 and F3 correspond to collected fractions and the asterisk indicates the collected fraction corresponding to SPINK2 protein. (B) Western blot against SPINK2 on three collected fractions F1, F2 and F3. Molecular weight is indicated on the left. The arrow represents SPINK2 signal.

**Figure S4: Protein sequence alignment of chicken acrosin (chicken ACR and ACRL proteins) with turkey (Mg), quail (Cj), horse (Ec), human (Hs), bovine (Bt), boar (Ss), mouse (Mm) and rat (Rn) acrosins.** No acrosin protein sequences could be retrieved from pubmed database in zefrafish and *Xenopus* species. The first residue of mature acrosins is indicated in red and the pro-peptide in blue (where appropriate). Identical and similar amino-acids conserved between species are indicated in black and grey, respectively. Protein sequences alignments were performed using Clustal Omega (http://www.ebi.ac.uk/Tools/msa/clustalo/) and amino acid identity/similarity were highlighted using BoxShade (http://embnet.vital-it.ch/software/BOX_form.html).
